# Work-related factors and hair cortisol concentrations among men and women in emergency medical services in Sweden

**DOI:** 10.1038/s41598-023-40076-x

**Published:** 2023-08-08

**Authors:** Anna M. Johnsen, Elvar Theodorsson, Anders Broström, Petra Wagman, Eleonor I. Fransson

**Affiliations:** 1 Department of Nursing, School of Health and Welfare, Jönköping University, Jönköping, Sweden; 2 Department of Biomedical and Clinical Science, Clinical Chemistry, Linköping University, Linköping, Sweden; 3 Department of Clinical Neurophysiology, Linköping University Hospital, Linköping, Sweden; 4 Department of Rehabilitation, Jönköping University, Jönköping, Sweden; 5 School of Health and Welfare, Jönköping University, Jönköping, Sweden

**Keywords:** Biomarkers, Endocrinology, Health occupations, Risk factors

## Abstract

Ambulance personnel in emergency medical services are exposed to physical demands and stress during work, and an increased prevalence of ill health has been observed in this group. The aim was to compare hair cortisol concentration (HCC) among Swedish ambulance personnel with HCC in a population-based reference sample, to analyse differences between women and men, and differences due to work-related factors. Samples of hair 1 cm closest to the skin (5–10 mg) were collected and analysed for cortisol by radioimmunoassay. Moreover, the participants responded to a questionnaire regarding their work environment. The HCC among the ambulance personnel did not differ from the HCC in the population-based reference sample (median 19.2 vs. 22.2 pg/mg, *p* = 0.319), nor were there statistically significant differences between women and men. Furthermore, no associations were found between HCC and physical and psychosocial work demands, work stress, or rest and recovery from work. However, occupational balance was positively correlated with HCC (r_p_ = 0.240; *p* = 0.044). The association remained statistically significant after adjustment for sex, age, hair bleaching, and corticosteroid treatment in a linear regression model. This study adds knowledge regarding HCC among ambulance personnel, and thus contributes to the overall picture of work environment and health for this group.

## Introduction

Previous studies indicate an increased prevalence of ill health among ambulance personnel, e.g., cardiovascular diseases^1^, musculoskeletal disease^1,2^, and mental health problems, including post-traumatic stress syndrome (PTSD)^3–6^, depression^7^, and anxiety^5^. The emergency medical services’ (EMS) work environment includes several factors that might harm the ambulance personnel’s health, e.g., long hours, shift work, heavy lifting, and work in awkward positions^8^. In addition to the physical workload, ambulance personnel are also exposed to acute and chronic stress during work^9^. There is still uncertainty regarding the cause of the high prevalence of health problems among ambulance personnel. One possible link might be the high exposure to acute and chronic stress during work, where repeated and prolonged stress activation might negatively affect the body^10^.

Acute stress exposure in the EMS includes facing critical incidents during work. Previous research has found that ambulance personnel rate events involving dead children, severe accidents or injuries, and acts of violence and threats as severely stressful^11^. Other examples of stressful critical incidents are taking care of severely injured or dying patients, driving under demanding situations, and handling the uncertainty of not knowing what will be encountered at the accident scene^8^. Stress of a more chronic nature in the EMS may be related to not knowing when the next emergency call will come, poor communication during work, lack of information, lack of support from colleagues and supervisors, and physical strain^11^. When comparing ambulance personnel and personnel in other health occupations, the ambulance personnel report more chronic stressors^11^, but also more emotional demands^11,12^.

The hypothalamus-pituitary-adrenal (HPA) axis is activated as a response to stress exposure. The HPA axis is, together with the autonomic nervous system, part of the body’s complex system to respond to stressful stimuli. When the HPA axis is activated, the hypothalamus secretes corticotropin-releasing hormone which stimulates the pituitary gland to secrete adrenocorticotropic hormone into the bloodstream, which in turn causes the adrenal glands to release the glucocorticoid cortisol into the bloodstream^13^. Cortisol has metabolic effects and by increasing blood glucose level, the body will be prepared to handle a stressful event. In addition, the HPA activation, together with the autonomic nervous response, will lead to haemodynamic changes e.g., increased blood pressure and increased heart rate. Moreover, the stress response activates the coagulation and immune systems^14^.

Today, the assessment of the individual stress response and activation of the HPA axis often includes measuring cortisol levels. The most used method is to measure cortisol in saliva, which gives an assessment of the stress level at a specific time. This method has previously been used in the EMS setting by Aasa et al.^15^, who measured cortisol in saliva during work and leisure time among ambulance personnel. They found higher morning cortisol levels among ambulance personnel with many health complaints compared to those with few health complaints. They also found associations between high morning cortisol and worries about work conditions.

Since cortisol in saliva represents the stress level at a specific time, this method restricts the possibility of drawing any conclusions regarding chronic stress exposure. A relatively new method of assessing exposure to stress over time is to measure the hair cortisol concentration (HCC)^16^. Since hair grows about 1 cm every month, it is possible to let the hair sample represent the stress exposure during a chosen period^17^. Previous research regarding the association between work demands and HCC is limited. However, Casjens et al.^18^ found higher HCC among shift workers in a manufacturing company during the COVID-19 pandemic compared to the pre-pandemic period. Manenschijn et al.^19^ found higher HCC among young shift workers compared to HCC among day workers. Moreover, van der Meij et al.^20^ found higher HCC among workers with high workloads, e.g., many work hours and numerous subordinates, compared to workers with a normal workload. To the best of our knowledge, there is limited research where HCC has been used to measure stress exposure in the EMS. However, one study by Behnke et al.^21^ found an association between high workload and high HCC among ambulance personnel. The often-stressful work situation in the EMS, which might be reflected in HCC, could be one link to the high prevalence of health problems among ambulance personnel. Moreover, the proportion of women has increased in the EMS during recent years^22^, why it is also important to understand how HCC differs between females and males. Therefore, this study aimed to compare the cortisol concentrations in hair among Swedish ambulance personnel with a population-based reference sample, and to analyse if there were differences between women and men or differences depending on work-related factors.

## Materials and methods

### Study design and setting

The data collection for this cross-sectional study was conducted between February and August 2017, in an EMS in the south of Sweden. The population served by the EMS was about 350,000, with a density of 34 per km^2^. In 2016, when the study was initiated, the total number of emergency calls was 45,600^23^. In 2017, approximately 170 employees were working in the EMS; about 70% were registered nurses (RN) and about 30% were emergency medical technicians (EMT).

### Participants and data collection

All permanently employed RNs and EMTs, engaged in work including patient care (n = 159), were invited to participate in the study. Of the invited ambulance personnel, 79 responded positively to the request to participate, resulting in a response rate of 50%. The data collection included hair samples and questionnaires. The hair was cut from the posterior vertex region of the head, close to the skin, by a group of staff employed in the EMS who had received specific training in the procedure. The posterior vertex region was used in accordance with the recommendation by the Society of Hair Testing^24^ since this region has the least variance in growth rate. Approximately 20 hair strands were cut close to the skin. From the cut sample, the 1 cm closest to the skin was cut, placed in a small plastic bag with a zip lock, and sent for analysis to the Division of Clinical Chemistry at Linköping University hospital. The participants were asked to report treatment for diseases such as psoriasis, asthma, arthritis, eczema, or the use of other drugs with corticosteroids. They were also asked about bleaching of the hair. The study was conducted in line with the principles of the Declaration of Helsinki^25^, and approved by the Regional Ethics Review Board of Linköping (Dnr 2016/482-31). Written informed consent was obtained from all the participants included in this study.

### Hair cortisol concentration analysis

Hair cortisol was extracted and analysed through a competitive radioimmunoassay (RIA). Hair samples were cut into small pieces. Each sample was put into a 2 mL QiaGenRB sample tube with a 0.5 mm QuiGen stainless steel bead. The samples were weighted on a Sartorious MC 210p microscale and homogenised for two minutes using a Retch Cryomill (20 Hz). 1 mL of the methanol was added to each tube and the samples were extracted overnight on a moving board. Afterwards, 0.8 mL of methanol supernatant was pipetted off and lyophilised using a Savant Speed Vas Plus SC210A. The samples were dissolved in radioimmunoassay buffer and analysed. The primary antibody used was Rabbit Cortisol 3 Polyclonal Antibody (MyBiosource, San Diego, USA). The secondary antibody which was anti-rabbit IgG was Sac Cell AA-Sac 1 (InmmunoDiagnostic System Ltd, Bordon, England). 16% of the samples from the SCAPIS study^26^ were analysed in the same week and using the same reagents and calibrators as the 79 samples from the present study in order to minimise the risk of between-assay bias influencing the conclusions of the study. Hair samples of between 5 and 10 mg were required to maintain a total inter-assay coefficient of variation below 8% for hair extraction and measurement of cortisol by the radioimmunoassay. The mean weight/mass of the 79 samples analysed was 9.6 mg, SD = 2.2. One of the samples weighed 3.9 mg. The method is fully described elsewhere^27^.

### Questionnaire

Previously used, well-established, and validated questionnaires regarding work-related factors^28–32^ were used. The questions regarding risk for accidents, threats and violence have previously been used by the occupational health service who served the EMS in this study. Moreover, one question regarding work stress was composed for this study.

The participants were asked about several characteristics regarding work, work schedule, having other work, and regarding their family situation.

Physical demands during work were evaluated using three questions^28^ regarding whether work required: more physical effort than standing and walking; lifting more than 15 kg several times a day; awkward or bending positions. The answers to each of the questions were reported on a six-point scale with the endpoints ‘not at all’ (1) and ‘almost all the time’ (6). The answers to the three questions were summarised, building an index ranging from 3 to 18, and categorised into low (3–6), medium (7–12), and high (13–18) physical demands during work. The risk for accidents during work was reported on a four-point scale, ranging from ‘absent’ to ‘high’, and dichotomised into low and high risk. Worries about threats and violence were reported on a four-point scale and dichotomised into ‘never/seldom’ and ‘sometimes/often’.

Psychosocial work demands, control, and support were evaluated using the 17-item Swedish Demand–Control–Support Questionnaire (DCSQ)^29^. The participants answered each item on a four-point scale, with the endpoints ‘no, almost never’ and ‘yes, often’. The items within each domain (demands, controls, and support) were summarised and dichotomised into low or high, using the median score in the current sample as a cut-off; demands (five items, median = 13), control (six items, median = 18), and support (six items, median = 19). Work-related stress in terms of job strain was defined as having high demands and low control according to the above categorisations. Work stress was also assessed using one general question, ‘Do you experience stress at work’. The response options were no, sometimes, or yes. Over-commitment^30^ was evaluated using three items, rated on four-point scales, with the endpoints ‘Strongly disagree’ and ‘Strongly agree’. The answers were summarised and dichotomised into high or low over-commitment, using the median score (median = 5).

Rest and recovery from work were assessed using a 14-item questionnaire^31^. The participants reported each item on a four-, five- or six-point scale. The answers were summarised into three indexes based on a principal component analysis performed in a larger study from which the participants in the present study represent a sample; recovered (five items), fatigue (four items), and sleep problems and worries (four items). One question ‘Do you feel energetic during a working day?’ was excluded since it loaded in all three components. The upper quartiles were used to dichotomise the participants into not enough recovery (≥ 12), high levels of fatigue (≥ 13), and high levels of sleep problems and worries (≥ 10). The participants’ perception of their mix of activities in everyday life (occupational balance) within and beyond paid work, was assessed using the 11-item Occupational Balance Questionnaire (OBQ11)^32^. The participants answered each item on a four-point scale, with the endpoints (0) ‘Completely disagree’, and (3) ‘Completely agree’. A total score (0–33) was generated by summing the items, and the median score was used to dichotomise the participants into low (< 16) or high (≥ 16) occupational balance.

### Population-based reference values for hair cortisol concentration

The HCC for ambulance personnel in this study was compared with population-based reference HCC values based on previously published results on the cohort from the Swedish CArdioPulmonary biolmage Study (SCAPIS) site in Linköping^26^. The SCAPIS was initiated as a national collaboration between six universities in Sweden aiming to reduce cardiovascular diseases, chronic obstructive pulmonary diseases, and related metabolic disorders. About 30 000 participants aged 50–64 years were included in the study^33^. In total 3156 participated in the HCC data collection, of whom n = 1247 were men and n = 2009 were women. The mean age among the participants was 57 (SD = 4)^26^.

### Statistical analysis

Background characteristics are presented as numbers and proportions, or means and standard deviations, within the total sample and within the sub-groups of women and men. Two-sided t-tests, chi-square tests, and Fisher’s exact tests were used to identify statistically significant differences between women and men. The Wilcoxon signed rank test was used to compare the HCC in the present study with the median HCC in the reference sample. Both the median HCC in pg/mg with interquartile range (IQR), and the mean HCC using the base-10 logarithm of the HCC are presented for subgroups with different work-related factors. Since the HCC was not normally distributed in this sample, the base-10 logarithm of the HCC was used when calculating Pearson’s correlation coefficients and comparing groups by one-way ANOVA and two-sided t-tests. In addition to the results presented in the results, non-parametric statistics regarding the associations between HCC and work-related factors were performed and can be found as Supplementary Table S1 online. The results from the non-parametric tests did not alter the interpretations or the conclusions in any way. Linear regression analyses were performed, using the base-10 logarithm of HCC, to estimate the strength of the association between HCC and occupational balance and to analyse the effect of other variables on this association.

To achieve a higher number of participants in the analyses, missing values were imputed in some of the index variables. The median score or the individual’s answers in the specific index were used as imputed values. No more than one missing value in each index was allowed when performing the imputation. In total, two subjects had imputed variables in recovery, three subjects in occupational balance, one subject in work demands, two subjects in work control, and one subject in work support.

The level of significance was set at 0.05. All statistical analysis was performed using IBM SPSS Statistics for Windows, version 27.

## Results

The background characteristics of the study population regarding age, occupation, work schedule, living conditions, and factors that might affect the HCC are presented in Table 1. The participants’ age ranged from 27 to 63 years, mean 47.7 years. There was no difference in age between women and men. The number of years working in the EMS ranged from 1 to 39 years, with a mean of 17.6 years, with statistically significantly more years among men than women (19.8 vs. 14.1, p = 0.023). Two-thirds of the participants were working 24-h shifts, and one-third were alternating 24-h shifts with day and night shifts. About one-quarter of the participants reported bleached hair, with a significantly higher proportion among women than men (63 vs. 2%, *p* < 0.001). One-fifth of the participants reported use of corticosteroid treatment. No associations were found between age and HCC. Hair bleaching or corticosteroid treatment did not affect the HCC in this study (Table 2). No differences in HCC were found between participants with different background characteristics e.g., occupation, work schedule, or living conditions (data not shown).

**Table 1:** Background characteristics of all the participating ambulance personnel and stratified by sex.

	Total n = 79	Women n = 28	Men n = 51	*p*
Age, mean (SD)	47.7 (9.2)	47.6 (9.4)	47.7 (9.2)	0.998^a^
Occupation, n (%)				
Registered nurses	68 (86)	26 (93)	42 (82)	0.311^c^
Emergency Medical Technicians	11 (14)	2 (7)	9 (18)	
Years working in EMS, n = 69, mean (SD)	17.6 (10.3)	14.1 (8.2)	19.8 (11.0)	**0.023**^a^
Work schedule, n (%)				
Day shift	3 (4)	0 (0)	3 (7)	0.349^c^
24-h shifts	47 (65)	20 (74)	27 (60)	
Alternating 24-h shifts with day and night shifts	22 (31)	7 (26)	15 (33)	
Having other work alongside the full-time ambulance work, n = 72, n (%)	22 (31)	5 (19)	17 (38)	0.086^b^
Hours per week, mean (SD)	6.1 (6.7)	4.2 (2.2)	6.6 (7.5)	0.498^a^
Living conditions, n (%)				
Living alone	11 (14)	5 (18)	6 (12)	0.516^c^
Cohabiting	66 (86)	23 (82)	43 (88)	
Children at home, n (%)				
Yes	41 (53)	15 (54)	26 (53)	1.000^b^
No	36 (47)	13 (46)	23 (47)	
Hair bleaching, n (%)				
Yes	18 (24)	17 (63)	1 (2)	**< 0.001**^b^
No	58 (76)	10 (37)	48 (98)	
Corticosteroid treatment, n = 78, n (%)	15 (19)	7 (25)	8 (16)	0.378^b^
Oral	3 (4)	3 (11)	0 (0)	**0.043**^c^
Inhaled	3 (4)	1 (4)	2 (4)	1.000^c^
External*	10 (13)	4 (14)	6 (12)	0.740^c^

^a^t-test.

^b^Chi^2^.

^c^Fisher’s exact test. Significant *p* values (< 0.05) are presented in bold.

Descriptive statistics using numbers and proportions or means and standard deviations. Tests of statistical differences between women and men.

*: One participant had both inhaled and external corticosteroid treatment.

**Table 2:** Associations between hair cortisol concentration (HCC) and some of the background characteristics among the ambulance personnel.

	n	HCC, median pg/mg (IQR)	Log10-HCC, mean (SD)	r*	p*
Age	79			− 0.134	0.240^a^
Sex					
Women	28	23.5 (11.6–47.0)	1.40 (0.41)		0.719^b^
Men	51	19.0 (14.4–32.2)	1.44 (0.42)		
Hair bleaching					
Yes	18	19.1 (14.3–34.6)	1.40 (0.43)		0.731^b^
No	58	24.9 (11.0–48.4)	1.44 (0.42)		
Corticosteroid treatment					
Yes	15	19.2 (12.4–72.5)	1.42 (0.40)		0.728^b^
No	63	21.3 (13.9–34.9)	1.46 (0.50)		

^a^Pearson correlations.

^b^t-test.

*IQR* interquartile range.

*: The base-10 logarithm of the HCC was used in the analysis.

**Figure 1:**
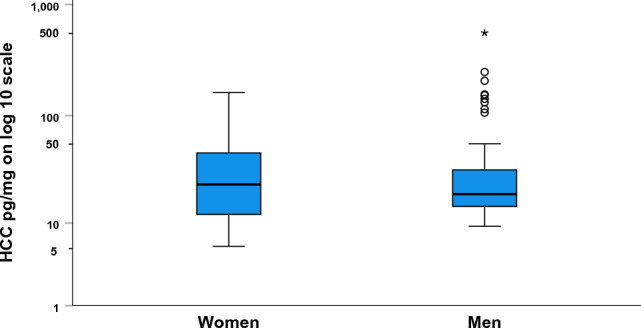
Hair cortisol concentrations (HCC) for women (n = 28) and men (n = 51). ○: Mild outlier > 1.5 times the interquartile range from quartile 3, *: extreme outlier > 3 times the interquartile range from quartile 3.

The comparison between the HCC among the participants in this study and the median HCC in the population-based reference sample from the SCAPIS is presented in Table 3. The HCC among the ambulance personnel in this study did not differ significantly from the median HCC in the SCAPIS. The HCC for the participating women and men in the present study is presented in Fig. 1. The median HCC did not differ significantly between women and men. However, the interquartile range was narrower (14.4–32.2 vs. 11.6–47.0), but with more outliers among the male participants.

**Table 3:** Comparison between hair cortisol concentration (HCC) among the participants in the present study and the population-based reference values from the SCAPIS-study^26^. The results are presented for all participants and stratified by sex.

	The present study		Reference sample from SCAPIS		*p*
	n		n		
HCC, median pg/mg (IQR)					
All participants	79	19.2 (13.9–34.9)	3156	22.2 (14.8–43.6)	0.319^a^
Women	28	23.5 (11.6–47.0)	2009	20.1 (13.6–38.2)	0.281^a^
Men	51	19.0 (14.4–32.2)	1147	25.9 (17.0–52.0)	0.090^a^

^a^Wilcoxon signed rank test.

*IQR* interquartile range, SCAPIS the Swedish CArdioPulmonary biolmage Study.

**Figure 2:**
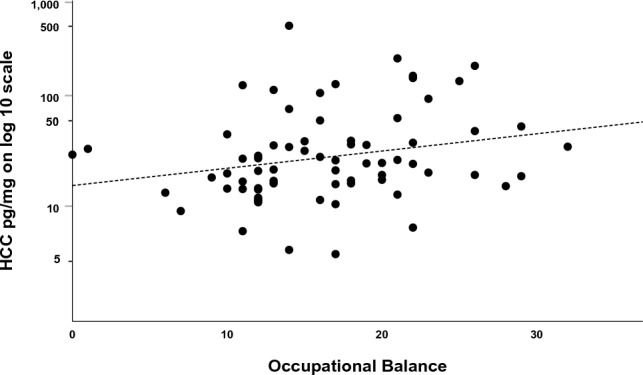
Scatterplot between hair cortisol concentration (HCC) among personnel in emergency medical service and occupational balance.

The associations between HCC and work-related factors are presented in Table 4. The results regarding physical and psychosocial demands, work stress, and over-commitment did not show any correlation to HCC. Rest and recovery from work in terms of recovery, fatigue, sleep problems and worries, did not correlate or show differences between groups. However, occupational balance was positively correlated with HCC (r = 0.240, *p* = 0.044), i.e. participants who reported better balance were observed to have higher HCC (Fig. 2). The corresponding non-parametric tests of the associations between HCC and work-related factors can be found in the Supplementary Table S1 online. However, these tests did not alter the results or the conclusions in any way.

**Table 4:** Associations between hair cortisol concentration (HCC) and work-related factors in the emergency medical service.

	n	HCC, median pg/mg (IQR)	Log10-HCC, mean (SD)	r*	p*
Physically demanding work	70			− 0.014	0.911^a^
Low	10	18.6 (15.2–33.0)	1.37 (0.27)		0.775^b^
Medium	31	23.9 (13.3–55.4)	1.45 (0.42)		
High	29	21.7 (12.2–31.9)	1.38 (0.43)		
Risk for accidents					
Low	27	19.0 (12.0–34.5)	1.38 (0.44)		0.568^c^
High	50	20.3 (14.3–41.1)	1.44 (0.39)		
Worries about threats and violence					
Never/seldom	46	19.1 (13.4–34.6)	1.40 (0.39)		0.675^c^
Sometimes/often	31	21.5 (13.9–48.2)	1.44 (0.43)		
Work demand	71			0.046	0.700^b^
Low	52	21.6 (14.6–34.8)	1.42 (0.40)		0.972^c^
High	19	17.1 (13.1–72.5)	1.42 (0.44)		
Work control	71			0.174	0.148^a^
Low	24	19.6 (12.3–38.6)	1.35 (0.35)		0.277^c^
High	47	21.7 (14.6–34.9)	1.46 (0.44)		
Work support	71			0.062	0.609^a^
Low	28	18.9 (14.7–34.5)	1.39 (0.33)		0.623^c^
High	43	23.7 (13.3–40.3)	1.44 (0.46)		
Job strain (high demands—low control)					
No	66	22.4 (14.4–41.1)	1.44 (0.42)		0.099^c^
Yes	5	14.4 (9.2–19.8)	1.13 (0.19)		
Work stress					
No	14	16.4 (11.9–41.1)	1.32 (0.31)		0.443^b^
Sometimes	46	19.1 (30.8–40.0)	1.44 (0.43)		
Yes	12	24.9 (14.8–93.5)	1.53 (0.44)		
Over-commitment	71			0.114	0.344^a^
Low	45	21.3 (14.5–32.8)	1.39 (0.38)		0.386^c^
High	26	25.5 (13.2–72.5)	1.48 (0.46)		
Recovery	70			0.058	0.635^a^
Enough	54	21.6 (14.6–37.0)	1.43 (0.39)		0.919^c^
Not enough	16	20.7 (12.6–37.5)	1.42 (0.49)		
Fatigue	70			− 0.090	0.460^a^
Low level	58	21.4 (14.6–41.1)	1.44 (0.42)		0.137^c^
High level	12	19.3 (9.7–30.9)	1.25 (0.29)		
Sleep problems and worries	71			− 0.067	0.579^a^
Low level	55	22.9 (15.3–40.3)	1.45 (0.40)		0.281^c^
High level	16	14.0 (10.9–29.3)	1.32 (0.45)		
Occupational balance	71			0.240	**0.044**^**a**^
High	38	23.0 (15.6–56.2)	1.49 (0.42)		0.124^c^
Low	33	17.6 (12.8–29.8)	1.34 (0.39)		

^a^Pearson correlations.

^b^One-way ANOVA.

^c^t-test.

*IQR* interquartile range.

*: The base-10 logarithm of the HCC was used in the analysis.

Significant *p* values (< 0.05) are presented in bold.

The result of the linear regression to estimate the strength of the association between occupational balance and HCC (Table 5), showed a statistically significant association (β = 0.016, *p* = 0.044) that remained statistically significant when adding sex and age to the model. The effect size was on the same level when adding hair bleaching and corticosteroid treatment; however, it did not reach statistical significance in this model. When adding work stress to the regression model, the effect size did not change in any substantial way (data not shown). A sensitivity analysis was performed, including only participants with complete data on all co-variates (n = 68) also in the crude model and model 1. The result was not statistically significant for any of the models; however, the effect size was comparable with the results in Table 5 (crude: β = 0.015; model 1: β = 0.014; model 2: β = 0.016).

**Table 5:** Multivariate associations between hair cortisol concentration (HCC) and occupational balance in the emergency medical service, β with 95% CI (Confidence Interval).

	Crude, n = 71		Model, 1 n = 71		Model 2, n = 68	
	β (95% CI)	*p*	β (95% CI)	*p*	β (95% CI)	*p*
Occupational balance	0.016 (0.000–0.031)	**0.044**	0.016 (0.000–0.032)	**0.047**	0.016 (− 0.002–0.033)	0.080
Sex						
Women			0		0	
Men			0.080 (− 0.119–0.279)	0.424	0.196 (− 0.098–0.491)	0.187
Age			− 0.001 (− 0.012–0.010)	0.900	− 0.001 (− 0.012–0.010)	0.832
Hair bleaching						
No					0	
Yes					0.152 (− 0.178–0.481)	0.361
Corticosteroid treatment						
No					0	
Yes					0.037 (− 0.248–0.322)	0.797

Model 1, adjusted for sex, and age. Model 2, additionally adjusted for hair bleaching, and corticosteroid treatment. The base-10 logarithm of HCC was used in the analyses.

Significant *p* values (< 0.05) are presented in bold.

## Discussion

The main findings in this study were that the HCC among ambulance personnel did not show any statistically significant difference from the HCC in a population-based reference sample and that no difference was found in the HCC between women and men among the ambulance personnel. Furthermore, we did not find any associations between HCC and physical and psychosocial work demands, work stress, or rest and recovery from work. However, the occupational balance was positively correlated with HCC.

The theoretical rationale for this study was the high prevalence of health problems among the ambulance personnel^1,5,7^, the possible link with work demands in the EMS, including both acute and chronic stressors^9^, and the possible activated HPA axis. We had hypothesised that the HCC among ambulance personnel would be increased compared to a reference sample. However, since the HCC among the ambulance personnel in the present study did not differ from the population-based reference sample in the cohort from SCAPIS^26^, it is not possible to draw any conclusions regarding the possible link between the activated HPA axis and the higher prevalence of health problems in this group. The high prevalence of health problems among ambulance personnel might still be due to acute and chronic stress, but maybe not through the HPA axis. Chandola et al.^34^ argued that stress might affect behaviour and lifestyle, e.g., food habits, leisure time physical activity, alcohol use, and smoking, through which the risk of cardiovascular disease might be increased. Moreover, the high prevalence of health problems in this group might be due to lifestyle factors^35^, without activation of the HPA axis.

The mean age differed between the participating ambulance personnel and the reference sample, with older age in the SCAPIS cohort (mean 57 vs. 48 years). Several previous studies have found that HCC increases with age^36–38^, but there are also conflicting results where no or negative associations between HCC and age have been found^36^. Given a positive association between HCC and age, this could hide a difference between the ambulance personnel and the reference sample. In the present study, the mean time for the personnel working in the EMS was 17.6 years. This long experience might affect how acute and chronic stress are handled by the personnel.

Moreover, there was no statistically significant difference in HCC between the ambulance personnel and the reference sample in the analyses stratified by sex. Results from previous studies regarding associations between HCC and sex, indicate increased HCC among men^36–38^. However, there are also conflicting results with no association between HCC and sex, or increased HCC among women^36^. The HCC in the reference sample is in line with previous research that has shown increased HCC among men. However, the HCC among the ambulance personnel indicates the opposite, with increased HCC among women compared to men (23.5 vs. 19.0 pg/mg). One possible explanation for this could be if the work in the EMS is more demanding^39^ and thereby more stressful for women compared to men. However, the observed difference in HCC is small and not statistically significant.

Behnke et al.^21^ found an association between high workload in the EMS and high HCC, an association which was not confirmed in this study. When comparing the results from these two studies, one important aspect is how the workload was defined. Behnke et al. ^21^ used a self-reported number of night shifts, number of medical rescue operations, and number of routine transportations one month before the assessment to define the individual workload. In the present study, the physical demands during work were defined by self-reported questions regarding physical effort, heavy lifting, and awkward or bending positions. The workload defined by Behnke et al.^21^ might include other dimensions of stress that are more related to the activation of the HPA axis than the physical demands assessed in the present study. Previous results regarding stressors in the EMS are based on self-reported questions^8,11^. However, there is limited knowledge regarding what causes an activation of the HPA axis among ambulance personnel.

No associations were found between psychosocial work demand, work control, work support, or the combination of high work demand and low work control i.e. job strain and HCC. These results are in line with a meta-analysis by Stalder et al.^38^, including 66 independent studies, where no associations were found between self-reported stress and HCC. In other studies where the association between work demand, control, support, and job strain have been assessed in relation to HCC, no significant associations have been found^20,40,41^. Based on the results from the present study and in accordance with previous research, self-reported work stress is not associated with an alteration in HCC.

This study showed that occupational balance was positively correlated with HCC, i.e. participants who had high ratings for balance had higher HCC. OBQ11 includes dimensions related to work and is therefore of interest in this study. High occupational balance has previously been associated with low stress symptoms^42^, while previous results regarding increased HCC have mostly been related to worse health status^26,43^, major life stressors^27^, or post-traumatic stress disorders^44^. The associations between high occupational balance and high levels of HCC in the present study are, therefore, somewhat contradictory and one possible explanation is that the association is due to a type 1 error. However, if a true association between HCC and OBQ11 exists among ambulance personnel, there are some explanations in which light this could be comprehended. The OBQ11 does not include questions regarding the number of activities or what these are specifically; instead, it assesses the participants’ perception of their total mix of different activities in life. To rate a high balance between activities could thus imply that they engage in many activities, both during work and leisure time, but the participants still experience a good mix of activities. Since we did not ask about the number of activities, no conclusions can be drawn, but one interesting aspect to consider is whether ambulance personnel have chosen the occupation based on the special work environment, including a variety of work tasks, and the uncertainty of never knowing what they will encounter during the next assignment. Granter et al.^45^ describes the ambulance occupation as an edgework and a high-hazard occupation, and moreover, that this could be attractive for the ambulance personnel. This could to some extent be in line with the discussion regarding the positive correlation between occupational balance and HCC, especially as Granter et al.^45^ argued that the work environment in the EMS has become more intense in many dimensions during recent years, leading to unnecessarily extreme conditions for the ambulance personnel.

## Strengths and limitations

Few studies have measured HCC among ambulance personnel, this study thereby adds important knowledge regarding the activation of the HPA axis for this group. Moreover, this study contributes knowledge about the weak association between self-reported work demands, both physical and psychosocial, and HCC, which also have been reported in other studies^20,38,40,41^. This is a study with a cross-sectional design. It is therefore not possible to draw any causal conclusions from the results. However, this study adds information to the overall picture concerning work environment and health for this group. When interpreting the results from this study it is important to be aware of the rather small sample size, which is a limitation in the study. A higher number of participants would have improved the statistical power of the analyses. It should be observed that several tests were performed, and that the observed findings might be due to type 1 error. A correction of the significance level, e.g. according to the method by Bonferroni, would have eliminated the association between HCC and OBQ11 in this study, but it would also have increased the risk of not detecting a true association, i.e., a type 2 error^46^. Moreover, a significant difference in HCC may not be of clinical significance for the individual participant.

An important aspect when interpreting the results is that the age of the participants in this study (27–63 years) differs from the reference sample from SCAPIS where the participants were older (50–65 years). A meta-analysis by Stalder et al.^38^ found a significant association between age and HCC, and comparing these groups may thereby introduce a risk of bias. As mentioned above, given a positive association between HCC and age, a true difference between the ambulance personnel and the reference sample could be hidden due to difference in age distribution. Moreover, the low response rate (50%) could to some extent be explained by the fact that many of the invited participants did not have any hair and were therefore not able to participate in this study. A strength of the study is that the hair samples were collected by trained staff, instead of the participants collecting the samples by themselves.

## Conclusions

The main finding in this study was that the HCC among ambulance personnel did not differ from the HCC in a population-based reference sample. Based on these results, HCC does not seem to be the link between high exposure to acute and chronic stress in the EMS and increased prevalence of health problems in this group. Moreover, there was no difference in HCC between women and men, nor were the work-related factors associated with HCC. However, a positive, somewhat unexpected correlation was found between occupational balance and HCC, which might be related to the complexity of demands and stressors in EMS. This is one of the first studies where HCC has been measured among ambulance personnel, shedding light on the potential link between work and health for this group.

### Supplementary Information

Supplementary Information.

## Data Availability

The dataset generated and analysed during the current study are not publicly available due to privacy and ethical restrictions. Requests regarding the data can be sent to the corresponding author.

## References

[1] Karlsson K, Nasic S, Lundberg L, Mårtensson J, Jonsson A (2022). Health problems among Swedish ambulance personnel: Long-term risks compared to other professions in Sweden—a longitudinal register study. Int. J. Occup. Saf. Ergon..

[2] Aasa U, Barnekow-Bergkvist M, Angquist KA, Brulin C (2005). Relationships between work-related factors and disorders in the neck-shoulder and low-back region among female and male ambulance personnel. J. Occup. Health.

[3] Hegg-Deloye S (2014). Current state of knowledge of post-traumatic stress, sleeping problems, obesity and cardiovascular disease in paramedics. Emerg. Med. J..

[4] Sterud T, Ekeberg Ø, Hem E (2006). Health status in the ambulance services: a systematic review. BMC Health Serv. Res..

[5] Wagner SL (2020). Ambulance personnel: Systematic review of mental health symptoms. Traumatology.

[6] Lozano R (2012). Global and regional mortality from 235 causes of death for 20 age groups in 1990 and 2010: A systematic analysis for the Global Burden of Disease Study 2010. Lancet.

[7] Reti, T., de Terte, I. & Stephens, C. Traumatic exposure, work-related stressors and gender as risk factors in the development of psychological distress for ambulance personnel. *Traumatology* (2021).

[8] Sterud T, Hem E, Ekeberg O, Lau B (2008). Occupational stressors and its organizational and individual correlates: A nationwide study of Norwegian ambulance personnel. BMC Emerg. Med..

[9] Donnelly E (2012). Work-related stress and posttraumatic stress in emergency medical services. Prehosp. Emerg. Care.

[10] McEwen BS (2002). Protective and damaging effects of stress mediators: The good and bad sides of the response to stress. Metabolism.

[11] van der Ploeg E, Kleber RJ (2003). Acute and chronic job stressors among ambulance personnel: Predictors of health symptoms. Occup. Environ. Med..

[12] Hansen Claus D, Rasmussen K, Kyed M, Nielsen K, Andersen J (2012). Physical and psychosocial work environment factors and their association with health outcomes in Danish ambulance personnel—a cross-sectional study. BMC Public Health.

[13] Tsigos C, Chrousos GP (2002). Hypothalamic–pituitary–adrenal axis, neuroendocrine factors and stress. J. Psychosom. Res..

[14] Kivimäki M, Steptoe A (2018). Effects of stress on the development and progression of cardiovascular disease. Nat. Rev. Cardiol..

[15] Aasa U, Kalezic N, Lyskov E, Angquist KA, Barnekow-Bergkvist M (2006). Stress monitoring of ambulance personnel during work and leisure time. Int. Arch. Occup. Environ. Health.

[16] Staufenbiel SM, Penninx BW, Spijker AT, Elzinga BM, van Rossum EF (2013). Hair cortisol, stress exposure, and mental health in humans: A systematic review. Psychoneuroendocrinology.

[17] Short SJ (2016). Correspondence between hair cortisol concentrations and 30-day integrated daily salivary and weekly urinary cortisol measures. Psychoneuroendocrinology.

[18] Casjens S (2022). Investigating the influence of shift work rosters on stress measured as cortisol in hair during the SARS-CoV-2 pandemic. Psychoneuroendocrinology.

[19] Manenschijn L, van Kruysbergen RGPM, de Jong FH, Koper JW, van Rossum EFC (2011). Shift work at young age is associated with elevated long-term cortisol levels and body mass index. J. Clin. Endocrinol. Metab..

[20] van der Meij L, Gubbels N, Schaveling J, Almela M, van Vugt M (2018). Hair cortisol and work stress: Importance of workload and stress model (JDCS or ERI). Psychoneuroendocrinology.

[21] Behnke A (2020). Associating Emergency Medical Services personnel’s workload, trauma exposure, and health with the cortisol, endocannabinoid, and N-acylethanolamine concentrations in their hair. Sci. Rep..

[22] Crowe RP (2020). Females and minority racial/ethnic groups remain underrepresented in emergency medical services: A ten-year assessment, 2008–2017. Prehosp. Emerg. Care.

[23] Nysam. *Ambulanssjukvård Nyckeltal 2016* (Helseplan Nysam AB, 2017).

[24] Cooper GAA, Kronstrand R, Kintz P (2012). Society of Hair Testing guidelines for drug testing in hair. Forensic Sci. Int..

[25] World Medical Association. WMA Declaration of Helsinki—Ethical principles for medical research involving human subjects (2013). 10.1001/jama.2013.281053 24141714

[26] Faresjö T (2020). Elevated levels of cortisol in hair precede acute myocardial infarction. Sci. Rep..

[27] Karlen J, Ludvigsson J, Frostell A, Theodorsson E, Faresjo T (2011). Cortisol in hair measured in young adults—a biomarker of major life stressors?. BMC Clin. Pathol..

[28] Alfredsson L (2002). Job strain and major risk factors for coronary heart disease among employed males and females in a Swedish study on work, lipids and fibrinogen. Scand. J. Work Environ. Health.

[29] Sanne B, Torp S, Mykletun A, Dahl AA (2005). The Swedish Demand—Control—Support Questionnaire (DCSQ): Factor structure, item analyses, and internal consistency in a large population. Scand. J. Public Health.

[30] Siegrist, J., Li, J. & Montano, D. Psychometric properties of the effort-reward imbalance questionnaire. Department of Medical Sociology, Faculty of Medicine, Duesseldorf University, Germany (2014).

[31] Gustafsson K, Lindfors P, Aronsson G, Lundberg U (2008). Relationships between self-rating of recovery from work and morning salivary cortisol. J. Occup. Health.

[32] Håkansson C, Wagman P, Hagell P (2020). Construct validity of a revised version of the Occupational Balance Questionnaire. Scand. J. Occup. Ther..

[33] Bergström G (2015). The Swedish CArdioPulmonary BioImage Study: Objectives and design. J. Intern. Med..

[34] Chandola T (2008). Work stress and coronary heart disease: What are the mechanisms?. Eur. Heart J..

[35] Zhang Y-B (2021). Combined lifestyle factors, all-cause mortality and cardiovascular disease: A systematic review and meta-analysis of prospective cohort studies. J. Epidemiol. Community Health.

[36] Wosu AC, Valdimarsdottir U, Shields AE, Williams DR, Williams MA (2013). Correlates of cortisol in human hair: Implications for epidemiologic studies on health effects of chronic stress. Ann. Epidemiol..

[37] Wester VL, Van Rossum EFC (2015). Clinical applications of cortisol measurements in hair. Eur. J. Endocrinol..

[38] Stalder T (2016). Stress-related and basic determinants of hair cortisol in humans: A meta-analysis. Psychoneuroendocrinology.

[39] Armstrong DP, Sinden KE, Sendsen J, Macphee RS, Fischer SL (2019). The Ottawa Paramedic Physical Ability Test: Test-retest reliability and analysis of sex-based performance differences. Ergonomics.

[40] Janssens H (2017). Hair cortisol in relation to job stress and depressive symptoms. Occup. Med..

[41] Qi X (2015). Relationship between work strain, need for recovery after work and cumulative cortisol among kindergarten teachers. Int. Arch. Occup. Environ. Health.

[42] Håkansson C, Lexén A (2021). The combination of psychosocial working conditions, occupational balance and sociodemographic characteristics and their associations with no or negligible stress symptoms among Swedish occupational therapists—a cross-sectional study. BMC Health Serv. Res..

[43] Manenschijn L (2013). High long-term cortisol levels, measured in scalp hair, are associated with a history of cardiovascular disease. J. Clin. Endocrinol. Metab..

[44] Van Den Heuvel LL (2020). Hair cortisol levels in posttraumatic stress disorder and metabolic syndrome. Stress.

[45] Granter E, Wankhade P, McCann L, Hassard J, Hyde P (2019). Multiple dimensions of work intensity: Ambulance work as edgework. Work Employ. Soc..

[46] Armstrong RA (2014). When to use the Bonferroni correction. Ophthalmic Physiol. Opt..

