# Supplementary material for: Work-related factors and hair cortisol concentrations among men and women in emergency medical services in Sweden
Source: Sci Rep. 2023 Aug 8;13:12877. doi: 10.1038/s41598-023-40076-x (PMC10409737; doi:10.1038/s41598-023-40076-x)
Supplement: Supplementary file 1 — Supplementary Information. [file 41598_2023_40076_MOESM1_ESM.pdf]

Supplementary Table S1

	n	HCC, median pg/mg (IQR)	r	p
Physically demanding work	70		-0.070	0.563[a]
Low	10	18.6 (15.2-33.0)		
Medium	31	23.9 (13.3-55.4)		0.791[b]
High	29	21.7 (12.2-31.9)		
Risk for accidents				
Low	27	19.0 (12.0-34.5)		0.426[c]
High	50	20.3 (14.3-41.1)		
Worries about threats and violence				
Never/ seldom	46	19.1 (13.4-34.6)		0.856[c]
Sometimes/often	31	21.5 (13.9-48.2)		
Work demand	71		0.009	0.941[a]
Low	52	21.6 (14.6-34.8)		0.645[c]
High	19	17.1 (13.1-72.5)		
Work control	71		0.122	0.313[a]
Low	24	19.6 (12.3-38.6)		0.388[c]
High	47	21.7 (14.6-34.9)		
Work support	71		0.058	0.630[a]
Low	28	18.9 (14.7-34.5)		0.809[c]
High	43	23.7 (13.3-40.3)		
Job strain (high demands – low control)				
No	66	22.4 (14.4-41.1)		0.062[c]
Yes	5	14.4 (9.2-19.8)		
Work stress				
No	14	16.4 (11.9-41.1)		
Sometimes	46	19.1 (30.8-40.0)		0.464[b]
Yes	12	24.9 (14.8-93.5)		
Over-commitment	71		0.038	0.755[a]
Low	45	21.3 (14.5-32.8)		0.599[c]
High	26	25.5 (13.2-72.5)		
Recovery	70		-0.059	0.628[a]
Enough	54	21.6 (14.6-37.0)		0.711[c]
Not enough	16	20.7 (12.6-37.5)		
Fatigue	70		-0.129	0.289[a]
Low level	58	21.4 (14.6-41.1)		0.207[c]
High level	12	19.3 (9.7-30.9)		
Sleep problems and worries	71		-0.229	0.054[a]
Low level	55	22.9 (15.3-40.3)		0.103[c]
High level	16	14.0 (10.9-29.3)		
Occupational balance	71		0.305	**0.010[a]**
High	38	23.0 (15.6-56.2)		0.068[c]
Low	33	17.6 (12.8-29.8)		

Table S1. Associations between hair cortisol concentration (HCC) and work-related factors in the emergency medical service.

IQR interquartile range. a: Spearman's correlations, b: Kruskal-Wallis test, c: Mann-Whitney U test, Significant p-values (<0.05) are presented in bold.
